# Endocranial volumes and human evolution

**DOI:** 10.12688/f1000research.131636.1

**Published:** 2023-05-30

**Authors:** Ian Tattersall

**Affiliations:** 1 Anthropology, American Museum of Natural History, New York, NY, 10014, USA

**Keywords:** human, hominin, evolution, brain expansion, endocranial volumes, cognition

## Abstract

Enlarging brains have been held up as the classic (if not the only) example of a consistent long-term trend in human evolution.  And hominin endocranial volumes certainly expanded four-fold over the subfamily’s seven-million-year history, while on a very coarse scale later hominids showed a strong tendency to have larger brains than earlier ones.  However, closer scrutiny of this apparent trend reveals that it was extremely episodic and irregular, a fact that argues against the notion that it was driven by social interactions internal to the hominin clade.  In addition, an overall tendency to brain volume increase was expressed independently and concurrently within at least three separate lineages of the genus *Homo* – suggesting that, whatever the exact influences were that promoted this global trend, they need to be sought among stimuli that acted comprehensively over the entire vast range of periods, geographies and environments that members of our subfamily occupied.  Significantly, though, the dramatic recent shrinkage of the brain within the species *Homo sapiens* implies that the emergence of modern human cognition (via the adoption of the symbolic information processing mode, likely driven by the spontaneous invention of language in an exaptively enabled brain) was not the culmination of the overall hominin trend towards brain enlargement, but rather a departure from it.

## Introduction

Hominin endocranial volumes, particularly as corrected for body size, have long held an iconic position in narratives of human evolution. Partly this is because the large brain of *Homo sapiens* is not only one of our species’ most conspicuous morphological characteristics, but is also one that is somehow associated with our most prized behavioral attribute, namely the unique way in which we think. And partly it is because endocranial volume, its close proxy, is both easily measurable and fairly readily available from fossils that represent nearly all chronological periods of human evolution. But perhaps above all, endocranial volume is iconic in paleoanthropology because it is one of the few hard-tissue hominin characteristics that seems to provide support for the existence among hominins of a long-term evolutionary trend of the kind Charles Darwin (1859) told us we should expect to find in the fossil record generally. This is important because, ever since the ornithologist Ernst Mayr sternly lectured us in 1950 that the human ecological niche was too broad to permit more than one hominin species to exist in the world at any one time, and that human evolution must perforce have consisted of the steady modification over time of a single lineage ( Mayr, 1950), paleoanthropologists have been mesmerized by a strictly gradualist notion of human evolution ultimately harking back to a time when a useful hominin fossil record barely existed (see Tattersall, 2009). This obsession is, conspicuously, not shared with other areas of vertebrate paleontology, in which the focus is nowadays typically upon how the rich taxic diversity of the living world was generated; and the unfortunate result of the insular paleoanthropological preoccupation with slow transformation has been a staunchly minimalist approach to hominin systematics.

**Figure 1.**
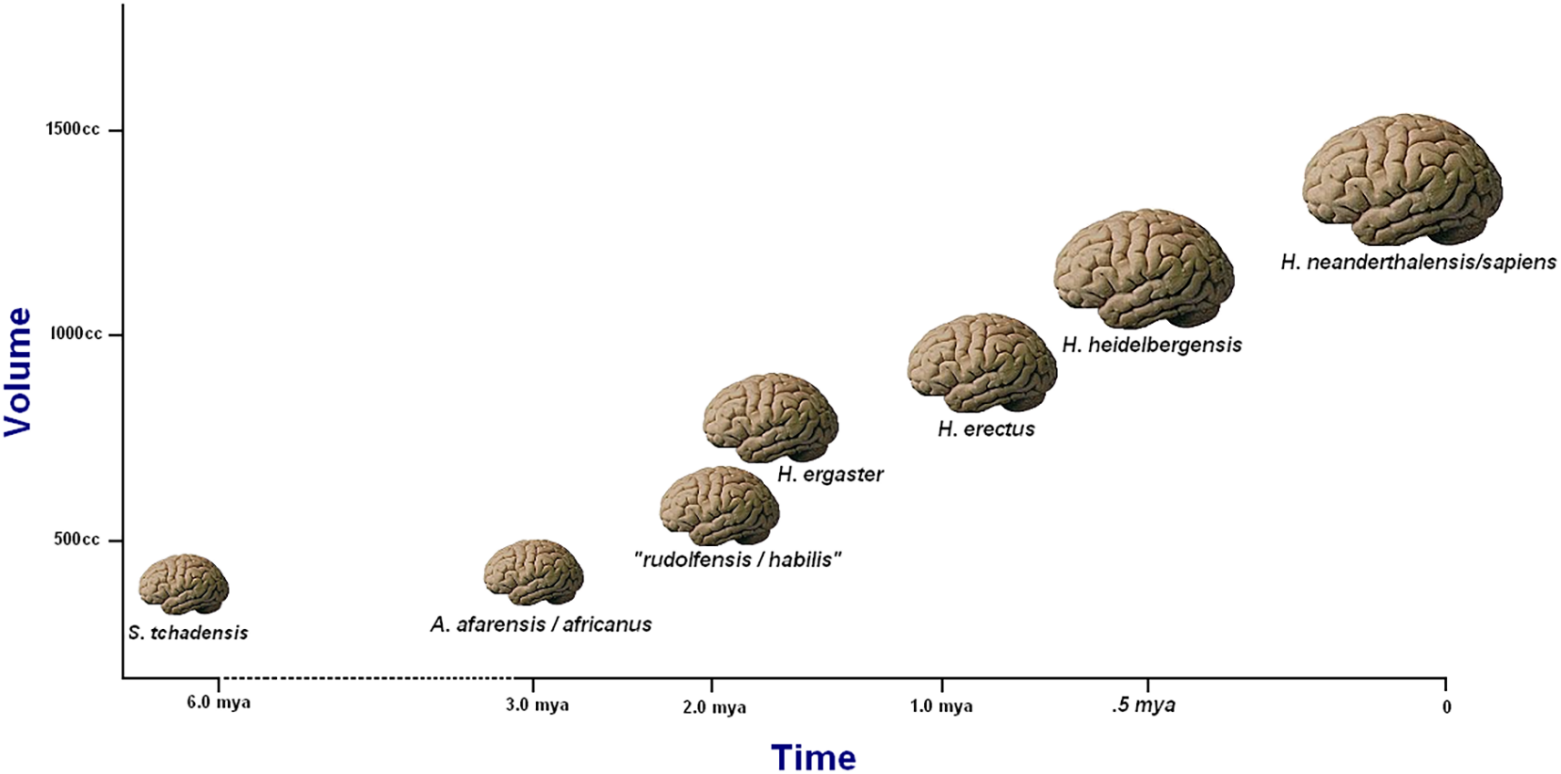
Fairly typical graphic rendition of the trend toward increasing hominin brain volumes with time. The trend is superficially accurate, but it suffers from an inadequate taxonomy and disguises a more irregular underlying pattern. From Tattersall (2008).

This minimalist bias, which favors stuffing the maximum number of fossils into the minimum number of analytical units (species), has been steadfastly maintained even as evidence has increasingly accumulated of a remarkably wide range of morphologies in the hominin fossil record ( Tattersall, 2022): a range that cannot plausibly be contained within any linear model of hominin evolution. Nonetheless, the linear perspective tenaciously persists; and no hominin feature has seemed to justify it more strongly than the apparently inexorable enlargement of the hominin brain ( Figure 1) that has taken place over the past few million years (if, indeed, there is such a thing as the hominin brain, in contradistinction to the brains of multiple species belonging to the hominin subfamily). On the face of it, the implication of steady brain size increase would appear to be that, as the millennia passed, “smarter” individuals simply out-reproduced “dumber” ones: a conclusion that might well seem self-evident to members of a species that prides itself above all on its “intelligence” (however one might care to measure that elusive quality). Yet it appears increasingly likely that the evolutionary message actually encoded in this averaged-out brain size expansion is entirely different.

## Evolutionary process and the fossil record

Before we ask whether or not the notion that our “intelligence” was gradually perfected over the eons has led us up a blind alley, we need first to inquire whether the reigning linear/minimalist expectation is itself realistic or not. And that means looking briefly at the nature of the evolutionary process that ultimately transformed a relatively unremarkable Miocene ape ancestor into the “know thyself” hominin of today. Incidentally, Linnaeus (1758) may have thought he was simply sidestepping an awkward issue by replacing an expected diagnosis of *Homo sapiens* with that rather cryptic exhortation, but his advice actually reflects directly on the uniquely explicit nature of modern human knowing.

By the middle of the twentieth century, anglophone evolutionary thought was pretty thoroughly dominated by a “hardened” ( Gould, 1983) version of the New Evolutionary Synthesis. This held that virtually all evolutionary phenomena could be ascribed to the gradual effects of natural selection within lineages, as environmental forces gradually honed the adaptations of each one. The lineage splitting that was self-evidently necessary to give rise to the biodiversity so glaringly characteristic of the living world was generally viewed as a special case of the same gradual process; and the general effect of this bias was the emphasis of gradual transformative change at the expense of splitting, as in the case of Mayr’s (1950) diatribe on the paleoanthropological record.

It took several decades for most paleontologists to realize that this description of the evolutionary process (or more accurately, processes) was actually a rather breathtaking oversimplification. Not only did it transpire that in the past environments had typically changed erratically, and on short timescales that would have rendered any adaptations rapidly obsolete, but it was also soon recognized that it was the fates of species as wholes, rather than of individuals within them, that most importantly determined macroevolutionary patterns. It is after all of little advantage in the long run to be the best-adapted member of your species in some respect, if that entire species is going extinct as a result of environmental change or because of new competition.

What is more, the individuals on whom classical natural selection is expected to act are complex bundles of overwhelmingly polygenic characteristics that are typically influenced by massively pleiotropic genes, so that the favoring by natural selection of one particular attribute will probably come at the cost of destabilizing others that may be equally or even more important to individual success. And whatever the molecular mechanism of change, it is clearly the entire organism that succeeds or fails in the ecological struggle, not its individual parts: a reality that makes it fruitless to try to follow the “evolution of the brain,” or the “evolution of the foot,” independently of the taxa in which those structures are embedded. Given all this, it is hardly surprising that, rather than acting as a powerful agent of gradual directional change, classical natural selection has emerged as more typically a force for homeostasis, trimming off the less fortunate extremes from populations that are thereby kept fit to participate in the unceasing struggle for ecological space (see Tattersall, 2022). In practical terms, this means that in seeking the sources of phylogenetic novelties and the agents of their natural triage, it makes most sense to look at whole species or populations, and at the competition that takes place among those larger-scale actors on the ever-shifting ecological stage.

Sadly, adopting this perspective only increases our operational difficulties. This is because we paleoanthropologists are the victims not only of a history that has largely eschewed the vital matter of systematic diversity in favor of linear schemes, but also of a particularly acute expression of the complications that always lurk when systematists attempt to recognize species in the fossil record. Those difficulties arise because none of the three key attributes of fossils (time, place, and morphology) is closely associated with speciation ( Tattersall, 1986); and the resulting uncertainties will inevitably be especially severe in the case of a very recently evolved and closely-knit group such as Homininae, in which both time scales and degrees of morphological differentiation will often be minimal. The unfortunate combination of history with operational impediments has led to the woeful absence of the reliable and readily testable phylogeny of our subfamily that we need to underpin our further inquiries. And while most paleoanthropologists still tend to dismiss concerns about the alpha taxonomy of the hominins as merely “quibbling about names,” it remains true that if you don’t know who the actors are, you will never understand the play. And almost certainly, the known human fossil record actually contains more species – more independent evolutionary actors – than we are currently able to recognize.

Fortunately, the underestimation of species diversity will not distort the overall form of a phylogeny as gravely as the spurious impression of branching that is imparted by excessive splitting ( Tattersall, 1992). Nonetheless, if we cannot accurately know how individual fossils should be properly classified, we will remain unable to determine either the morphological or temporal limits of the species to which they belong; and if our bias is towards underestimation, we will end up with a smaller number of recognized taxa that, at the extreme, might approximate uncomfortably to a linear pattern, particularly if we are unduly influenced by how ancient we believe the fossils concerned to be. In paleoanthropology, the received linear perspective has encouraged us to see fossils essentially as chains running through time, which in turn has deeply influenced their nomenclature and classification – despite the fact that morphology alone has a necessary connection to phylogenetic relationship ( Eldredge and Tattersall, 1975). The unpalatable fact that we are working with an inadequate phylogeny thus severely limits what we can say about the history of any single and inherently variable morphological attribute – in this case, endocranial volume – in human phylogeny. Nonetheless, it is clear that our inherited attitudes toward human evolution have been a source of important source of distortion in our perceptions of the process by which we *Homo sapiens* became the entirely unprecedented cognitive entity we are today.

## Endocasts and archaeology

Where not credited otherwise, the brain volumes (and species averages) cited below are taken from Holloway *et al*. (2004). These values were obtained from the water displacement of physical endocasts (which often required reconstruction prior to measurement). More recently, neurocranial volumes have more commonly been obtained by digital extraction from CT scans (see Weber, 2014), a procedure that also commonly requires varying degrees of reconstruction. Occasionally the two methods produce significantly different results; for example, the Ngawi calvaria from Java was estimated to be 870 ml by water displacement ( Holloway *et al*., 2004), and 959 ml by digital extraction ( Kaifu *et al*., 2015). In general, however, it is probable that errors in volume estimation are likely to be more heavily influenced by the condition of the individual specimen than by the measurement method used.

Raw brain sizes are sometimes converted into Encephalization Quotients (EQs) by calculating the ratio of the measured brain volume to expected body volume. However, this fairly simple expedient actually introduces a variety of complications, not least among them the choice of the appropriate regression for deriving the expected value. Additionally, in the case of measured fossil crania, a) body size is usually unknown, and b) it is not possible to closely determine the relative volumes of different brain regions, let alone such physiological features as neuron densities. And while there are numerous potentially informative convolutions and fissures on the surface of the brain, paleoneurologists actually remain the most disputative members of the already highly fractious profession of paleoanthropology.

These numerous sources of uncertainty mean that neurocranial endocasts have yet to come anywhere close to providing us with even the most basic information on brain function and “intelligence” that we would ideally wish to have when addressing the evolution of human cognition. However, when we are seeking the origins of the unique modern cognitive style the biological record gives us nowhere else to turn; and few would contest that brain volume must mean *something*, especially when it can be related in some way to body size. It is an element that can never be forgotten, even when its exact cognitive implications are impossible to know. The resulting uncertainty makes it fortunate that we also have the archaeological record, the material testament to ancient human behaviors subsequent to the invention of stone tool making. Because, as frustratingly selective and incomplete as it is, this record does enable us to glimpse some ancient human cognitive styles at work.

## Brain volumes in early human evolution

**Figure 2.**
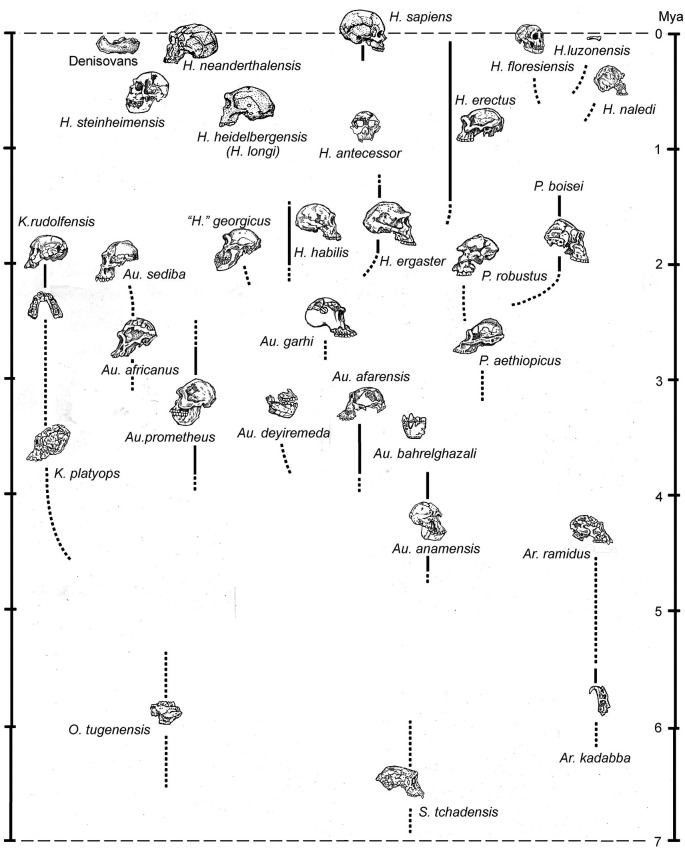
A very generalized family tree of the hominins, most importantly showing the tendency to diversify that, prior to the emergence of *Homo sapiens*, assured the typical coexistence of multiple hominin species at particular point in time. From Tattersall (2022).

The earliest putatively hominin species yet documented in the fossil record are represented by a rather motley and generally fragmentary assortment of African remains that are attributed to four species and dated to between about seven and four Ma (million years ago). Only two of those very early species furnish any information on endocranial volume, and one alone is well enough known to support even a guess about encephalization. The earlier of them, *Sahelanthropus tchadensis* from Toros-Menalla in the central-western African Republic of Chad, dates from around 7 Ma ( Brunet *et al*., 2002; Lebatard *et al*., 2008) and is the most ancient hominoid yet proclaimed to be hominin ( Figure 2). Virtual reconstruction of the single cranium available yielded an endocranial volume estimate of 360–370 ml ( Zollikofer *et al*. 2005), well below the modern chimpanzee average of 405 ml. Unfortunately, the affinities of a supposedly associated partial postcranial skeleton remain robustly debated ( Macchiarelli *et al*., 2020; Daver *et al*., 2022), although on skull size it seems reasonable to suppose that the individual’s body weight may have been toward the lower end of the very broad (30–60 kg) chimpanzee range. The other relevant “early hominin” species is *Ardipithecus ramidus* ( White, Suwa and Asfaw 1994, 1995), a much later form known from a handful of eastern African sites and dated to between about 4.5 and 4.3 Ma. The cranial volume of the one reasonably complete individual of this species is reported to be in the region of 300–350 ml ( White *et al*., 2009), implying a rather modest degree of encephalization (plausibly comparable to that of *Sahelanthropus*), since at about 50 kg her body weight was plausibly within the chimpanzee range ( Lovejoy *et al*., 2009). All in all, then, the little we currently know about the brains of the most ancient hominins supports the idea that in this early period hominin encephalization, and by extension “intelligence,” compared a little unfavorably with that of today’s great apes, which recent studies have shown to be remarkably sophisticated creatures (e.g., Krupenye *et al*., 2016).

Not a great deal had apparently changed in respect of brain size by the time the “australopiths” (genera *Australopithecus* and *Paranthropus*) appeared on the scene after about 4.2 Ma (see Table 1). That is, at least, the implication of the one cranium we know of the earliest australopith species, *Australopithecus anamensis.* This has an estimated endocranial volume of around 370 ml ( Haile-Selassie *et al*., 2019), closely akin to that of *Sahelanthropus.* If the australopiths as currently conceived are a genuinely monophyletic group (not a sure bet), brain enlargement, or even a tendency towards it, was evidently not one of its founding apomorphies.

**Table 1.** Mean endocranial volumes, with ranges and sample sizes, for hominin species for which data are available.

Species	Mean volume	Range	Sample size
*Sahelanthropus tchadensis*	360–370 ml		1
*Ardipithecus ramidus*	300–350 ml		1
*Australopithecus anamensis*	~370 ml		1
*Australopithecus afarensis*	446 ml	387–550 ml	4
*Australopithecus garhi*	450 ml		1
*Australopithecus aethiopicus*	432 ml	400–490 ml	4
*Australopithecus boisei*	508 ml	475–545 ml	6
*Australopithecus robustus*	493 ml	450–530 ml	3
*Australopithecus africanus* *^1^*	461 ml	400–560 ml	9
*Australopithecus sediba*	420 ml		1
*Kenyanthropus rudolfensis*	788 ml	752–825	2
“ *Homo habilis*”	609 ml	509–687	6
*Homo ergaster* ^2^	825 ml	750–900 ml	4
*Homo erectus* ^3^	959 ml	780–1,225 ml	15
*Homo heidelbergensis* ^4^	1,227 ml	1,165–1325 ml	6
*Homo steinheimensis* ^5^	1,227 ml	1,057–1,436 ml	15
*Homo neanderthalensis*	1,415 ml	1,172–1,740 ml	25
*Homo sapiens* (Pleistocene)	1,499 ml	1,285–1,775 ml	29
*Homo sapiens* (contemporary)	1,330 ml	1,250–1,730 ml	>500
*“Homo” naledi* ^6^	512 ml	465–560 ml	2
*“Homo” floresiensis* *^6^*	426 ml		1
“ *Homo” georgicus* ^7^	640 ml	546–775 ml	5

Data from Holloway *et al*. (2004), or as specified in the text.

^1^ Includes *A. prometheus*.

^2^ Turkana only, see text.

^3^ Java and China only, includes Ngandong.

^4^ Europe and Africa only, including China yields a mean of 1,248 ml.

^5^ Provisional nomenclature.

^6^ Misclassified as *Homo*.

^7^ Misclassified as *Homo*, arbitrarily includes all Dmanisi hominins.

It is only with the reported mean endocranial volume of the much better-known (and briefly coeval: approximately 3.9 to 3.0 Ma) *Australopithecus afarensis* (446 ml) that we find a noticeable inflection in the fossil record. That mean value, which is fairly close to those of the other non- *anamensis* australopith species in the 3.9 to 1.5 Ma time range ( Table 1), represents a volumetric increase of around 20 percent relative to *Sahelanthropus* and *Ardipithecus*, and an excess of about nine percent relative to the chimpanzee average of 405 ml. What was happening within the australopith radiation to promote and metabolically sustain this upward tick in brain size (and encephalization, given that body weights were likely within the general chimpanzee range) is hard to say, and there is certainly no very clear correlation with time. For example, whereas the earlier “robust” species *Paranthropus aethiopicus* has a lower mean endocranial volume (432 ml) than the later *P. boisei* (515 ml) and *P. robustus* (493 ml), the single value of 420 ml reported for the relatively recent (2 Ma) “gracile” species *A. sediba* is below the adult range reported for its earlier close relative *A. africanus* (although the endocast itself apparently shows more “humanlike” orbitofrontal proportions: Carlson *et al*., 2011).

What might be even more puzzling, especially to anyone given to linear thinking, is that it is hard to associate brain size with what was arguably the most consequential of all cognitive innovations in human evolution. This was, of course, the invention and manufacture of Mode 1 stone tools, a practice that by the standards of the day made extreme cognitive demands, and that was introduced by one, or perhaps more, australopith species in the period before about 2.8 Ma ( Plummer *et al*., 2023). Besides being demonstrably beyond the behavioral range of today’s great apes, this new behavior would clearly have done a lot to promote the leap in dietary quality that brain enlargement metabolically required. However, especially if the slightly larger brain volumes of the later robust species were themselves related to greater body weights, this fateful innovation is conspicuously unassociated with any concomitant or subsequent brain size increases among the australopiths. And the single measurable individual of the 2.5 Ma species *A. garhi*, while presumptively associated with early cut-marked bones ( De Heinzelin *et al*., 1999), has an endocranial volume of 450 ml, basically identical to the 446 ml mean ( Table 1) of its earlier close relative *A. afarensis.*

In Leakey *et al*. (2001), Meave Leakey and colleagues created the new genus and species *Kenyanthropus platyops* for a crushed but distinctively flat-faced 3.5 Ma hominin cranium found to the west of Lake Turkana in northern Kenya. Its endocranial volume could not be precisely measured but was estimated to lie in the australopith range. The specimen’s describers also called attention to resemblances with the much later (1.9 Ma) and larger-brained (752 ml) KNM-ER 1470 specimen from the other side of the lake, which many researchers were by then allocating to *Homo rudolfensis.* If, as seems likely, the two fossils do indeed belong to the same clade distinct from the australopiths, in this case we can justify perceiving a modest trend toward neurocranial enlargement over time.

## Brain volumes in the genus *Homo*

Our lingering fealty to Ernst Mayr’s (1950) linearity has assured the persistence of a conventional wisdom whereby all hominin fossils other than those classified as *Kenyanthropus, Paranthropus* or “earliest hominins,” must belong either to *Australopithecus* or *Homo.* This makes the allocation of a fossil to either genus essentially a matter of exclusion (“it’s not *Australopithecus*, so it must be *Homo*,” and vice versa). And it is certainly the only plausible explanation for the inclusion in the genus *Homo* (see for example Lordkipanidze *et al*., 2013) of the 1.9 Ma individuals from the Caucasian site of Dmanisi, the earliest hominin fossils we have from outside Africa. These have no clear morphological claim to membership in any genus that is defined by *H. sapiens* ( Schwartz *et al*., 2014); and indeed, they constitute a rather heterogeneous assemblage that is united principally by small brain volumes averaging 640 ml (range 546–775 ml, n = 5). This is admittedly an unremarkable set of values for any hominin of their period, and it is only marginally greater than the australopith range. Diagnosis by exclusion is even more evident in the cases of the remarkably recent, small-bodied, and small-brained hominin species dubbed *H. floresiensis* ( Brown *et al*., 2004) and *H. naledi* ( Berger *et al*., 2015). The former is dated to around 100 thousand years ago (Ka) and has a single endocranial value of 426 ml ( Kubo *et al*., 2013); the latter dates from around 300 Ka ( Dirks *et al*., 2017) and has brain sizes ranging from 465 to 560 ml ( Garvin *et al*., 2017, n = 2). Most probably, the distinctive hominin lineages represented by these two species primitively maintained relatively low brain to body size ratios. But whatever the exact reality may be, we have absolutely nothing to gain (in terms of understanding brain size trends, or anything else) by shoveling any of the hominins just discussed into the genus *Homo* just because they are patently not *Australopithecus.* Until the alpha taxonomy here has been properly sorted out, these fossils are better omitted from any discussion of brain size trends within the genus *Homo.*

Until Louis Leakey, Phillip Tobias and John Napier described *Homo habilis* from Tanzania’s Olduvai Gorge some 60 years ago ( Leakey *et al*., 1964), the concept of “early *Homo*” was exemplified by *Homo erectus* from Trinil (endocranial volume 940 ml), Sangiran (mean: 917 ml, range 813–1,059 ml, n = 5) and Ngandong (mean: 1,148 ml, range 1013–1231 ml, n = 6) in Java, and Zhoukoudian in China (mean: 1,046 ml, range 915–1,225 ml, n = 4). All were thought at the time to date from the early Middle Pleistocene, and all comfortably exceeded Arthur Keith’s (1948) 750 ml “cerebral Rubicon” for the achievement of “humanity,” by which he broadly meant membership in the genus *Homo.* And although Leakey and colleagues’ new hominin was the presumptive manufacturer of the Mode 1 stone tools found at Olduvai (hence the name), its allocation to the genus *Homo* was and remains entirely problematic. Just for a start, it was dated to a then-staggering 1.8 Ma ( Leakey *et al.*, 1961), right at the beginning of the Pleistocene; and among its type materials were two partial braincases that had estimated volumes of 687 and 650 ml., only very modestly out of the australopith range.

Unsurprisingly, then, general acceptance of *Homo habilis* came only a decade later, as a result of discoveries in northern Kenya’s Turkana Basin by researchers who shared Leakey’s agenda (see discussion in Tattersall, 2009). This acceptance required entirely disassociating membership in our genus from any rational appraisal of morphology, and the floodgates were opened. A motley assortment of fossils dating from over 2 Ma to around 1.5 Ma have now been assigned to *Homo habilis*, with the six available cranial volumes estimated at between 582 and 687 ml. Those who still regard the *Kenyanthropus rudolfensis* specimens as belonging to *Homo habilis* would augment this range with two additional endocranial volumes: 752 and 825 ml. However, the two endocranial volumes recorded for the coeval *K. rudolfensis* are both well above anything attributed to *H. habili*s. This not only strongly supports the notion of multiple taxa in this assemblage, but also suggests a stronger tendency to brain expansion in the *Kenyanthropus* lineage than we see in the rump attributed to *Homo habilis.*

The species *Homo erectus* is defined by the 940 ml Trinil skullcap, which is of very distinctive morphology and is now plausibly dated to around 800 Ka ( Hilgen *et al*., 2023). With this specimen we can very generally associate the slightly older or approximately coeval Sangiran (1.3–1.0 Ma, 917 ml) and Zhoukoudian (770 Ka, 1,046 ml) fossils, and the much younger and larger (ca. 110 Ka, 1148 ml) Ngandong materials (possibly of a different but closely related species: Schwartz and Tattersall, 2000), plus the crania from nearby Sambungmacan (probably 500 Ka or slightly younger; mean: 986 ml, range 917–1,035 ml, n = 3). Among the Javan specimens, at least, there appears to be a time-transgressive trend: crania from the Sangiran Dome and Trinil, the earliest sites, show the lowest volumes overall; the temporally intermediate Sambungmacan fossils exhibit an intermediate mean value; and the much younger Ngandong specimens are substantially larger yet. It should be noted that there is overlap in the ranges up the Javan succession, and that the Zhoukoudian specimens appear on average more voluminous than those of comparable age from Java; but within the clade as a whole, the temporal trend toward enlargement is nonetheless striking.

During the 1970s a succession of startling eastern African discoveries was made of hominin fossils that dated between <2.0 and 1.5 Ma and that showed larger brains than almost anything previously known from the time. After a period of limbo as *Homo* sp., these fossils were attributed to “early African *Homo erectus*” (e.g. Leakey and Walker, 1976). More reasonably, many would now assign them to the species *Homo ergaster* ( Groves and Mazak, 1975); but even so, they make up a very unwieldy assemblage, and it would almost certainly be highly misleading to cite a single endocranial mean for everything that has been called *Homo ergaster* or “early African *Homo erectus.*” Early in this period (1.78–1.6 Ma) the Mode 2 stone tool making tradition appeared; but beyond some degree of refinement of handaxe manufacture over time there is subsequently very little evidence of other conceptual advances.

The first two crania East African crania to be referred to *Homo erectus* or *H. ergaster* were the East Turkana KNM-ER 3733 and 3883 calvariae ( Leakey and Walker, 1976, both ca. 1.6 Ma: 848 and 804 ml., respectively). The endocranial volume of the KNM WT-15000 “Nariokotome Boy” cranium from West Turkana (1.5–1.6 Ma), described a decade later, is a broadly comparable 900 ml. But while the three crania are close in age, size, and geography, the morphological differences among them are striking. Even more remarkably, to one degree or another the same observation holds true for other eastern African crania generally assigned to this group. Those heterogeneous fossils include the KNM-ER 42700 calvaria from Ileret in East Turkana (~1.55 Ma, 721–744 ml: Spoor *et al*., 2007; its tiny volume may be at least partially attributable to slightly subadult status: Baab, 2008); the Olduvai OH 9 fossil (1.2–1.4 Ma, 1067 ml), the earliest known hominin with a cranial volume in excess of 1000 ml; the Daka calvaria NME BOU-VP 2/66 from Ethiopia (~1 Ma, 995 ml), the Buia cranium UA 31 from Eritrea (~1 Ma, 995 ml: Bruner *et al*., 2016); and the Olorgesailie partial calvaria KNM OG 45500 from southern Kenya (~900 Ka, 700–800 ml: Potts *et al*., 2004). In this rather motley assortment of fossil hominins it is hard to detect any consistent trend in endocranial volume over time. Indeed, if anything there is a tendency for later clearly adult individuals to have smaller brains. On current evidence it is impossible to say to what extent that effect might be due to non-randomly sampled smaller body sizes. But while this morphologically heterogenous eastern African assemblage of specimens almost certainly harbors representatives of multiple lineages (the temporal and morphological limits of which cannot be guessed), if as some believe just the single species *Homo ergaster* was involved, it was evidently a species that essentially showed stasis in brain volumes rather than any tendency toward expansion over its long (800 kyr) time span.

Where we do get a signal of brain enlargement is with the arrival of the species *Homo heidelbergensis.* This hominid had already appeared in both Africa and Europe by around 600 Ka, and it seems to have been gone by about 200 Ka (maybe rather less in eastern China: see below). However, dating for many important specimens remains very approximate. Exactly how the distinctive new lineage emerged from within the medley of earlier hominins is unclear; but with a mean endocranial volume of 1,227 ml and a range of 1,165–1,325 ml for the European Arago, Ceprano, and Petralona fossils plus the African Bodo, Kabwe, and Saldanha crania (a greatly reduced sample compared to that used by Holloway *et al*., 2004), there is no doubt that members of the new species boasted significantly larger brains than ever before. This brain expansion, and the cognitive advance it implies, does correlate broadly with a significant inflection in the archaeological record: it is during the tenure of *Homo heidelbergensis*, for example, that for the first time we encounter evidence of throwing spears, hafted tools, constructed shelters, Mode 3 tools, and so forth.

*Homo heidelbergensis*, which is also known from fossils found in eastern Asia (Dali, possibly ~550 Ka, 1,120 ml: Wu, 1981; Jinniushan, ~260 Ka, 1,330 ml: Rosenberg *et al*., 2006; and possibly Harbin [aka *H. longi*], 146 Ka, 1,420 ml: Ni *et al*., 2021), was the first cosmopolitan human species. And very generally, despite generally poor age controls, it appears that its later representatives tended to have larger brains than the earlier ones, a tendency particularly apparent in China, especially if the Harbin cranium is properly attributed to *H. heidelbergensis.* What is more, as apomorphic as its known representatives may be, many believe (in the absence of any better fossil alternatives) that in some general sense *H. heidelbergensis* was the ultimate source of later humans. If (a big “if”) this is correct, in Europe and western Asia some early and very plesiomorphic version of *H. heidelbergensis* gave rise, without any detectable change in average brain size, ( Table 1) to the lineage represented by the 448 Ka ( Demuro *et al*., 2019) hominins from the Sima de los Huesos in Spain’s Atapuerca Massif (mean 1,233 ml, range 1,057–1,436 ml, n=14: Poza-Rey *et al.*, 2019). In good linear fashion, the Sima hominins were initially assigned to the (morphologically very distinct) *H. heidelbergensis*; but their describers have since relented, admitting them to a separate lineage ( Arsuaga *et al*., 2014) that foreshadowed *Homo neanderthalensis.* The Sima hominins have not yet been formally given their own species name; but pending closer scrutiny of the matter one might provisionally call them *H. steinheimensis*, applying the name borne by the rather later (<350 Ka) Steinheim cranium from Germany with which they share some Neanderthal-like aspects of facial morphology. The endocranial volume of the German fossil was, however, estimated by Prossinger *et al*. (2003) to be around 1,140 ml which, although just within the lower limit of the Sima sample, is considerably below its mean.

*Homo neanderthalensis* itself was well established in Europe by about 175 Ka (Biache St Vaast: Guipert *et al*., 2011, ca 1200 ml), or even considerably earlier ( Stringer and Hublin, 1999) if the English Swanscombe braincase (1,325 ml) is properly attributed to this species. Before its extinction after about 40 Ka, this hominin left behind extensive archaeological and fossil records. Over the years, Neanderthal fossils have been rearranged into various morphs; but the species itself was highly distinctive, and the mean endocranial value of 25 adult Neanderthal individuals from all parts of its temporal and geographical distributions is 1,415 ml ( Table 1; the values used are taken from Holloway *et al*., 2004, but do not include all individuals identified as Neanderthal therein), with a range of 1,172 to 1,740 ml. Earlier Neanderthal individuals tended to have lower endocranial volumes than later ones; but the temporal trend within the species is not clear-cut. Within the larger Neanderthal clade, the major events we can infer evidently involved substantial morphological innovation; quite likely, very rapid increases in mean endocranial size heralded the appearances of both *H. steinheimensis* and *H. neanderthalensis.*

It is clear that *Homo sapiens* originated in Africa. And, purely for want of a better alternative, we can again surmise that its lineage originated in some plesiomorphic early population of *H. heidelbergensis.* The thankfully now largely defunct category of “Archaic *Homo sapiens*” (which embraced forms as various as the Ndutu partial cranium, ~350 Ka and ~1,100 ml: Rightmire, 1983; the Ngaloba LH 18 cranium, 120 Ka and 1,200 ml: Magori and Day, 1983; the Florisbad face and frontal (aka *H. helmei*), ~260 Ka: Grün *et al*., 1996, and somewhere between 1,280 and 1,450 ml: Bruner and Lombard, 2020; and the Jebel Irhoud 1 and 2 crania, ~300 Ka: Richter *et al*. 2017, and 1,375 and 1,467 ml respectively: Neubauer *et al*. 2018) helped divert attention from a remarkable morphological diversity among African members of the genus *Homo* in the latter part of the Pleistocene; but what is perhaps even more noteworthy is that at present there is nothing in the fossil record that seems to anticipate the highly apomorphic structure of the modern human skull and postcranial skeleton (see Tattersall and Schwartz, 2008). Unlike the Neanderthals, then, modern humans as yet have no known immediate fossil precursors. Nonetheless, we can very safely surmise that the notably large-brained earliest fossil *Homo sapiens* from Africa, including the Ethiopian Omo 1 (230 Ka: Vidal *et al*., 2022, but unfortunately too fragmentary for exact assessment of brain size), and Herto 1 crania (~160 Ka and ~1,450 ml: White *et al*., 2003), were ultimately descended from much smaller-brained forerunners. In other words, something like the trend toward increasing brain volume we have noted happening independently in eastern Asian *Homo erectus* and in the European Neanderthal lineage, must also have occurred autonomously in Africa as well.

Exactly what it was that propelled the metabolically expensive but common trend among discrete hominin lineages spanning a great range of time, geographies, and environments, is currently anybody’s guess – although it is hugely tempting to associate the brain size expansion involved with a generalized increase in “intelligence” that we see also reflected in the complexifying archaeological record. It would not, however, explain the appearance of the qualitatively different symbolic cognitive mode that we see in *Homo sapiens* today. Or the remarkable decline in human endocranial volumes that seems to have occurred since the late Pleistocene.

## Brain volumes and cognition in *Homo sapiens*

Modern *Homo sapiens* processes information in what is almost certainly an entirely unique fashion that is qualitatively distinct from even its most direct precursors. We modern humans deconstruct our surroundings and experiences into a vocabulary of discrete mental symbols that we can shuffle around, according to rules, to make statements about the world not only as it is, but as it *might* be (see Tattersall, 2012). Unlike other organisms, which live more or less directly in the worlds with which nature presents them, we live for most of the time in the worlds that we reconstruct in our heads. This cognitive innovation is the basis of the formidable planning ability that has enabled us so rapidly and unprecedentedly to eliminate all of our hominin competition, and that also accounts both for the explicit nature of human knowledge and the unprecedented impact (intended or otherwise) that our species has had on the planet that (so far) supports it (see discussion in Tattersall, 2012). Counterintuitively, though, the adoption of the symbolic algorithm was followed by a significant reduction in human endocranial volume ( Table 1). The mean volume of 29 Upper Paleolithic *Homo sapiens* braincases listed by Holloway *et al*. (2004) is 1,499 ml (range: 1,285–1,775 ml). This value is somewhat greater than the overall Neanderthal mean of 1,415 ml, although it is below the mean of 1,567 ml (range: 1,305–1,740 ml) for ten “Classic” and late eastern Neanderthals taken from the same list. Effectively, then, we can reasonably conclude that Neanderthals and Pleistocene *Homo sapiens* had brains of effectively the same size, probably even relative to body size. But the same source gives a mean value of 1,330 ml (range: 1,250–1,730 ml) for a sample of over 500 modern *Homo sapiens*, a value that represents an apparent reduction in average brain size of some 12.7 percent since the early days of our species.

This is an abrupt reversal of what had been a long-term tendency within the genus *Homo*, so to what might we attribute this striking reduction? I have reviewed the various available explanations ( Tattersall, 2018), and have concluded that the observed overall brain size reduction is most plausibly ascribed to the adoption of the symbolic cognitive algorithm. If so, symbolic information processing must have turned out to be more metabolically frugal than its “brute force” intuitive predecessor with the result that, whereas the quality of intuitive processing scaled generally with overall brain size, symbolic processing required a lesser overall volume of brain tissue to produce a superior cognitive result. This processing change would have required substantial prior innovation in the internal reorganization of the human brain, innovation that is most reasonably associated with the radical developmental alteration implicit in the structural changes that produced the highly autapomorphic *Homo sapiens* ( Tattersall, 1998, 2017). The anatomical modifications that announce the arrival of our own species are visible today only in the preserved hard tissues; but they are widely distributed throughout the skeleton, and the ontogenetic reorganization they reflect must surely have had ramifications in the soft tissues as well, including the nervous system. What exactly those changes might have been is beyond my remit here; but they evidently had to do largely with the refinement and multiplication of internal signaling pathways, as well as with the differential expansions of the brain structures associated with globularization and discussed by Neubauer *et al*. (2018).

Evidence from the Omo Basin in Ethiopia indicates that the new species *Homo sapiens*, and by extension its new biology, was already in existence by around 230 Ka ( Vidal *et al*., 2022). But current archaeological evidence suggests that the switch to symbolic information processing, along with its behavioral consequences, were not expressed until rather later, at some time around ( Tattersall, 2012), or somewhat before ( Marean, 2015), about 100 Ka. If so, whereas the enabling biology was acquired exaptively at the origin of *Homo sapiens*, modern cognition itself is a slightly later acquisition: an emergent potential based on anatomical innovation that had to be “discovered” behaviorally, much as ancestral birds only very belatedly discovered they could use their feathers to fly. The behavioral stimulus involved was almost certainly the spontaneous invention of language – which is, after all, the ultimate symbolic behavior, and one that could easily have been acquired in the short-term event ( Senghas *et al*., 2004; Berwick and Chomsky, 2016) that is all the record gives time for. The new way of communicating, and the cognitive capacity of which it was both cause and effect, would have spread fast among members of a species who already shared a “language-ready” brain. This would in turn have opened the way for the rapid spread of *Homo sapiens* within and beyond Africa, along with a whole panoply of unprecedented behaviors that made the new species competitively insuperable (see discussion in Tattersall, 2012). The earliest *Homo sapiens* were, then, still working on the old intuitive cognitive algorithm that made brain enlargement beneficial. But once symbolic information processing had been established, larger-than-necessary brains evidently became a metabolic liability. And average cranial volumes decreased, even as *Homo sapiens* was becoming the dominant component of terrestrial ecosystems worldwide.

## Conclusion

Its recent shrinkage notwithstanding, the major overall signal in the long history of the hominin brain was undoubtedly one of remarkably rapid enlargement over time. The earliest hominins had brains of around 350 ml in volume, whereas at its high point in the late Paleolithic our species *Homo sapiens* boasted a mean of 1,499 ml: a striking four-fold increase over a period of some seven million years. What is more, on a very coarse scale there is a strong and undeniable tendency for later hominins to have had larger brains than earlier ones. But it is nonetheless clear that the precise pattern of endocranial expansion (even as obscured by an inexact taxonomy) was not the smooth and gradual one we might expect to find if the trend toward greater brain size had been governed by social or other within-species processes. Remarkably often, within the overall picture of expansion we find patterns of stasis on both regional and Old World-wide scales, and instances in which current taxonomy is a disappointing predictor of brain volume.

The very early hominins had brains (and by implication brain:body size ratios) that were somewhat smaller than those of today’s great apes, although they were presumably comparable to those of their hominoid contemporaries. What small brain sizes may have meant in cognitive terms (theory of mind, for example) is impossible to specify; but given the phylogenetic position of these hominins they are hardly surprising. Odder, perhaps, is that the earliest species of *Australopithecus, A. anamensis*, boasted a brain comparable in size to that of the very much older *Sahelanthropus tchadensis*, and very much smaller than those of the later *Australopithecus* species which were indeed enlarged relative to the modern ape benchmark. To what influences we can attribute the substantial brain size difference between *A. anamensis* and the later but slightly overlapping *A. afarensis*, is as obscure as the cause of the relative stasis that we see subsequently among the other australopiths despite the introduction of the first stone tools at some time over 2.6 Ma. Most authorities reckon that, for energetic reasons, this hugely consequential cultural innovation was prerequisite to the later expansion that occurred among species of the genus *Homo* (which is itself generally supposed to have emerged from within the australopith group); but the availability of cutting tools apparently had no effect on australopith brain sizes. The fact that substantial brain enlargement did occur within the *Kenyanthropus* lineage between 3.5 and 1.9 Ma (if indeed this is a true lineage) makes one wonder if we might be looking for the antecedents of *Homo* in the wrong place.

Among the most striking morphological features of the fossils generally allocated to “early *Homo*” (apart from the anomalous *H. floresiensis* and *H. naledi* which, although misallocated to *Homo*, are at least on the face of it good examples of stasis) is a significant increase in brain size at the base of the lineage. In insular eastern Asia the record starts with *Homo erectus*, in which modestly-sized braincases consistently yield to increasing endocranial volumes over the period between >1 ma and 0.1 Ma. This contrasts with what we see approximately concurrently in Africa, where analysis is complicated by the lack of an adequate systematic structure, but where the endocranial volumes available demonstrate absolutely no consistent tendency towards enlargement in the period between about 2 Ma and 900 Ka. It is only with the arrival of *Homo heidelbergensis* at around 600 Ka that we find another apparent leap in African endocranial volumes. This is followed by another period of taxonomic uncertainty, at the end of which there had been further net brain size increases in the African lineages that resulted in *Homo sapiens* on the one hand, and the Jebel Irhoud hominin on the other. In Europe, the large Sima de los Huesos sample possessed a mean endocranial volume virtually identical to that of European and African *Homo heidelbergensis*; but within a very few hundred thousand years their presumed descendant *Homo neanderthalensis* was boasting a brain that was on average around 15 percent larger.

The major implication in all of this is that most, if not all, increments in hominin endocranial volume tended to coincide with the arrival of new species. There is relatively little suggestion, except possibly in insular eastern Asia, of any clear-cut intraspecies trends (and these fade if the Ngandong fossils are indeed their own species). So we have strong support for two different notions: first, that we will never properly understand the biological history involved without an adequate taxonomy; and second, that the driver of hominin brain size increase is more plausibly to be sought in inter-species than in intra-species interactions. And, while over the long haul the signal of brain size increase within the genus *Homo* is a very powerful one, that tendency seems to have been expressed independently in Africa, Europe and Asia, suggesting that its driver was something common to *Homo* populations in all periods, areas and environments. What exactly that driver might have been remains unknown, although it must have been related in some way to the apparently equally episodic cognitive and behavioral complexification we see reflected in the archaeological record.

What none of this will ever explain, however, is the emergence of the modern human symbolic style. This represents a radical departure from anything that preceded it, and it was clearly not just a refinement of an existing cognitive architecture. The archaeological record tells us emphatically that our recently extinct fossil relatives were extremely complex beings, capable of a wide array of behaviors that we once thought were unique to us; but up to now it has furnished no unequivocal evidence that any hominin other than *Homo sapiens* ever routinely exhibited symbolic behaviors. Evidently, it is possible to be extremely refined and accomplished, both cognitively and behaviorally, without being symbolic. There is clearly more than one way to be a really smart hominin. What is more, in *Homo sapiens* the adoption of symbolic cognition (which was necessarily enabled by a pre-existing – and exaptive – neural capacity) was followed by a significant decrease in overall brain size. That final step in human brain evolution was clearly based on both a history and governing circumstances that were unique to the *Homo sapiens* lineage; and, although built on earlier brain enlargement, it occurred entirely independently of the factors responsible for the striking but very irregular increase in cranial capacity that had previously dominated hominin evolution.

## Data Availability

No new data are associated with this article.
