# Supplementary material for: Endocranial volumes and human evolution
Source: F1000Res. 2023 May 30;12:565. [Version 1] doi: 10.12688/f1000research.131636.1 (PMC10517302; doi:10.12688/f1000research.131636.1)
Supplement: Peer review history

# Reviewer report: Reviewer response for version 1

**DOI:** 10.5256/f1000research.144497.r205817

**Published:** 2023-09-22

**Authors:** Pasquale Raia

**Affiliations:** 1 Department of Earth Sciences, Environment and Resources, Universita degli Studi di Napoli Federico II, Naples, Campania, Italy

Tattersal assembled a beautifully written, well-balanced, and informative review about what we know, and almost equally importantly what we don’t know, about the evolution of brain size (actually endocranial volume) in Homo. The author makes a fantastic job in debunking the simplistic idea – which is very common among non-experts- that endocranial volumes increased almost linearly during human evolution. More to the point, he clearly shows that there is a noticeable gap between what most people understand as early Homo (that is up to erectus included) and heildebergensis. He also very clearly points out that our understanding of the tempo and mode of endocranial evolution in Homo is made difficult by the absence of a clear, well-understood alpha taxonomy and phylogeny. Over the last chapter, the author replicates his older tenet that symbolic thought and language (the earnest evidence of ‘mentalese’) sets apart morphologically early Homo sapiens as found in Omo valley to the behaviorally modern humans appeared around 100ka.

 The paper is timely, easy to read and very informative review of the current knowledge on endocranial volume evolution, whether or not the author is correct in assigning to language the emergence of modern brain functioning, and to globularity its external manifestation.

Is the topic of the opinion article discussed accurately in the context of the current literature?

Yes

Are arguments sufficiently supported by evidence from the published literature?

Yes

Are all factual statements correct and adequately supported by citations?

Yes

Are the conclusions drawn balanced and justified on the basis of the presented arguments?

Yes

Reviewer Expertise:

Anthropology, evolutionary biology

I confirm that I have read this submission and believe that I have an appropriate level of expertise to confirm that it is of an acceptable scientific standard.

# Reviewer report: Reviewer response for version 1

**DOI:** 10.5256/f1000research.144497.r175885

**Published:** 2023-06-29

**Authors:** Dominique Grimaud-Herve

**Affiliations:** 1 Histoire Naturelle de l'Homme Préhistorique, CNRS UMR 7194, Département H&E, Museum National d'Histoire Naturelle, Paris, Île-de-France, France

This article is a synthesis of the data and results concerning the morphological evolution of the brain in relation to the cultural evolution of hominins, well referenced and argued. All species of the genus *Homo*, such as *Australopithecus*, *Kenyanthropus* and *Paranthropus* are reviewed. Individual value or average (when it comes to several specimens in the same deposit) of their endocranial volume of the species is considering, related to their chronological age, which allows the author to evoke and argue the possible origin of human groups coming from Africa as the migration routes to Asia and Europe. The latest discoveries of fossils, news species or results are discussed, argued and taken into account or not.

 The question of the relationship between the increase in cranial capacity and more efficient cognitive capacities is raised to be confronted with the contradiction of the decrease in endocranial volume since the end of the Pleistocene. The author presents few hypotheses to explain this reduction of cranial volume without really concluding, what we cannot blame him for, this subject of the relationship between the aspects: - functional and therefore the performance of the required task and - metabolic, i.e. the amount of energy required without becoming a handicap, is always still the subject of discussion in the scientific community.

 One of the results which highlights the independence of the evolutionary trend according to the continents is interesting and joins that observed on the skulls of which it has been shown that the species adapted to the environmental conditions of their geographical area, without being able to go further, in biological, cognitive or behavioral explanation or interpretation. The acquisition of symbolic thought, followed by a decrease in the overall size of the brain therefore remains unexplained, just because no study or analysis has yet come to fruition.

 In conclusion, this article is a good synthesis of the state of the question, which summarizes and analyzes the results of the bibliography, including that of the author who proposes several hypotheses to explain the arrival of symbolic behavior in *Homo sapiens*, and then the decrease in cranial capacity just after this cultural acquisition. The reviewer suggests evoking the case of the SH whose skeletons at the bottom of the pit were discovered with a magnificent biface in rose quartz never used and which could be interpreted as an offering and therefore a symbolic behavior?

 Few comments :

- Chronological age of Dmanisi hominins is 1.8 et not 1.9 (Lorkipanidze *et al.*,2013 ^1^)
- In the very often cited publication of Holloway (2004), which is a general synthesis of the work of many researchers, it would be preferable to quote the author of the research and the results obtained for each of the specimens to enhance their work.
- In figure 1, rather than putting a brain of HS of different sizes depending on the species, replace it with a drawing of the endocranium of each species? In effect, the cerebral form is not the same according to the species of hominins. This seems to reinforce the misconception that human evolution is linear. Appropriate drawings showing the different position of the cerebral lobes relative to each other according to the species, and therefore their cerebral reorganization, would better support the idea of ​​an arboreal evolution?

Is the topic of the opinion article discussed accurately in the context of the current literature?

Yes

Are arguments sufficiently supported by evidence from the published literature?

Yes

Are all factual statements correct and adequately supported by citations?

Yes

Are the conclusions drawn balanced and justified on the basis of the presented arguments?

Yes

Reviewer Expertise:

Paleoanthropology, paleoneurology

I confirm that I have read this submission and believe that I have an appropriate level of expertise to confirm that it is of an acceptable scientific standard.

## References

[1] : A complete skull from Dmanisi, Georgia, and the evolutionary biology of early Homo. *Science* .2013;342(6156) : 10.1126/science.1238484 326-31 10.1126/science.1238484 24136960
